# A time series driven decomposed evolutionary optimization approach for reconstructing large-scale gene regulatory networks based on fuzzy cognitive maps

**DOI:** 10.1186/s12859-017-1657-1

**Published:** 2017-05-08

**Authors:** Jing Liu, Yaxiong Chi, Chen Zhu, Yaochu Jin

**Affiliations:** 1 Key Laboratory of Intelligent Perception and Image Understanding of Ministry of Education, Xidian University, Xi’an, 710071 China; 2 Department of Computer Science, University of Surrey, Guildford, GU2 7XH UK

**Keywords:** Gene regulatory networks, Fuzzy cognitive maps, Big data, Big optimization, Multi-agent genetic algorithm, Decomposition

## Abstract

**Background:**

Reconstructing gene regulatory networks (GRNs) from expression data plays an important role in understanding the fundamental cellular processes and revealing the underlying relations among genes. Although many algorithms have been proposed to reconstruct GRNs, more rapid and efficient methods which can handle large-scale problems still need to be developed. The process of reconstructing GRNs can be formulated as an optimization problem, which is actually reconstructing GRNs from time series data, and the reconstructed GRNs have good ability to simulate the observed time series. This is a typical big optimization problem, since the number of variables needs to be optimized increases quadratically with the scale of GRNs, resulting an exponential increase in the number of candidate solutions. Thus, there is a legitimate need to devise methods capable of automatically reconstructing large-scale GRNs.

**Results:**

In this paper, we use fuzzy cognitive maps (FCMs) to model GRNs, in which each node of FCMs represent a single gene. However, most of the current training algorithms for FCMs are only able to train FCMs with dozens of nodes. Here, a new evolutionary algorithm is proposed to train FCMs, which combines a dynamical multi-agent genetic algorithm (dMAGA) with the decomposition-based model, and termed as dMAGA-FCM_D_, which is able to deal with large-scale FCMs with up to 500 nodes. Both large-scale synthetic FCMs and the benchmark DREAM4 for reconstructing biological GRNs are used in the experiments to validate the performance of dMAGA-FCM_D_.

**Conclusions:**

The dMAGA-FCM_D_ is compared with the other four algorithms which are all state-of-the-art FCM training algorithms, and the results show that the dMAGA-FCM_D_ performs the best. In addition, the experimental results on FCMs with 500 nodes and DREAM4 project demonstrate that dMAGA-FCM_D_ is capable of effectively and computationally efficiently training large-scale FCMs and GRNs.

**Electronic supplementary material:**

The online version of this article (doi:10.1186/s12859-017-1657-1) contains supplementary material, which is available to authorized users.

## Background

In the age of big data, there is an urgent need to develop effective and computationally efficient methods to convert data into knowledge. When we extract knowledge from big data, we often need to solve big optimization problems, which may involve thousands of, even millions of, decision variables. For example, in the “*Optimization of Big Data Competition*” organized at the *IEEE 2015 Congress on Evolutionary Computation*, a set of big optimization benchmark problems were formulated, which have up to 4864 decision variables extracted from EEG data analyses [1, 2].

In biology, techniques of high-throughput experiment such as DNA microarrays bring the “big data” in molecular biology and system biology, which is made up with a large number of molecular entities and their products. Finding the interactions between these entities is a key step to understand the governing mechanism of biological systems. The so-called gene regulatory networks, or GRNs in short, has been proved to be the most widely used model to analyze the dynamic behavior of biological systems. GRNs model the molecular entities as networks which consist of a group of nodes (representing genes, proteins and small molecules) which influence each other. And the objective of GRNs is to capture the interconnections among these genes. By reconstructing the complex interconnections within these GRNs, we can highlight inhibitory or excitatory interactions, as well as how intracellular or extracellular factors (environmental and drug-induced effects) affect gene products or deregulate cellular process. The reconstruction of a GRN based on expression data is also called reverse engineering or network inference.

In recent years, various algorithms, especially evolutionary algorithms (EAs) have been proposed to infer GRNs by analyzing of gene expression data, such as genetic algorithm (GA) [3], genetic programming (GP) [4], evolution strategy (ES) [5], and ant colony optimization (ACO) [6]. However, the GRNs modeled by the above algorithms only consist of a limited number of genes. How to reconstruct the large scale GRNs is still an underdetermined problem in biology.

Various models have been used to model GRNs. The simplest models are based on Boolean networks. In the reverse engineering, Boolean networks are used to infer both the underlying topology and the Boolean functions at the nodes from observed gene expression data [7].

In addition, continuous networks, an extension of the Boolean networks, are also widely used to model GRNs. Each gene is considered to be a node in the network, while the edge represents the relationship between genes. In biological systems, the activity level of genes is considered in a continuous range, continuous representation captures this feature, while the Boolean model is not work. Many methods based on continuous networks have been proposed to the inference of GRNs. Such as linear regression based, and the mutual information (MI) based methods. ARACNE algorithm [8], the classical MI based method, calculate the value of MIs of all gene pairs. If the calculated value exceeds the given threshold, then one regulatory interaction is inferred [9].

Many probabilistic graphical models have been proposed by researchers to measure the high-order dependency among distinct gene expression patterns. The Bayesian network is one of the most popular methods used in the inference of GRNs. In the Bayesian network, directed acyclic graphs are used to indicate the conditional dependency among random variables [10].

Despite that plenty of proposed models using gene expression data to infer GRNs [11], these models only adopt scaled real values or Boolean values to indicate the levels of gene expression [12]. FCMs use fuzzy values, which integrate the benefits of both real values and Boolean values, to figure out the real relationships in GRNs [12]. Many researches [13] have validated that FCMs are efficient and powerful tool when it comes to modeling complex regulatory network systems.

Thus, within this context, we focus on developing a new evolutionary approach framework to train GRNs, which is based on fuzzy cognitive maps, or FCMs in short. As a type of effective computation tool, fuzzy cognitive maps (FCMs) were proposed by Kosko in [14] for the first time. Many work have been demonstrated the effective ability of FCMs in modeling complex systems. An FCM is a network model to describe the relationship between different concepts in the real world. Nodes in the network stand for the concepts and weighted edges are used to quantify the relationship between concepts. Compared to traditional modeling techniques, FCMs are more reasonable and intuitive description of human reasoning and have been successfully used in numerous practical application areas, such as medical diagnosis [15], metabolism network modeling [16], process control [17], military science [18], and modeling of software development [19, 20].

In recent years, lots of work has been carried out in the research of train FCMs from data. In fact, given an initial state of an FCM, which is represented by a set of state values of constituent nodes, a trained FCM can simulate the evolution of state values over time to predict the future state values. Thus, the objective of these learning algorithms is to determine weights in FCMs so that the response time sequences of each node in the trained FCMs can fit the observed time sequences as far as possible.

Therefore, viewed from methodology, the learning objective of reconstructing FCM models from time series data can be described as an optimum formula. And the learning algorithm is to minimize the optimum formula as far as possible, which is simulating the observed time sequence. This will become a typical big optimization problem when the scale of FCM expands to a high level, since the number of decision variables, namely, the number of weights needs to be determined, increases quadratically with the number of nodes in FCMs. For example, if we need to train an FCM with 500 nodes, 250 000 decision variables need to be optimized is a typical large-scale optimization problem.

Evolutionary algorithms (EAs) are a subset of generic population-based optimization algorithms inspired by the biological evolution. In real applications, EAs often performs well to a wide range of problems. EAs and other population based metaheuristics have been shown to be powerful in training FCMs, including genetic algorithms (GAs) [21, 22], particle swarm optimizations (PSOs) [23], simulated annealing (SA) [24], ant colony optimization (ACO) algorithms [6, 25], and memetic algorithms [26].

Stach et al. [21] proposed a method to optimize FCMs using real-coded genetic algorithm (RCGA). The aim of RCGA is to model the system structure from the time sequence which was observed from the complex system. Since time series data only at two adjacent time point can be used as a training sample, the whole time sequence can be broken down into several pairs according to time point *t* and *t* + 1. The estimated values of time point *t* + 1 can be calculated by the values of time point *t* in each pair. The objective of learning algorithm is to find a FCM that can reproduce the time sequence which is the same as the observed time sequence. The RCGA first established the error between the output data of estimated FCM and the observed time sequence as an optimum formula, then, the operators of RCGA were used to solve the optimum formula which is actually minimize the error. The experiments results in [21] show that RCGA have a big advantage over ES.

Subsequently, in order to train large scale FCMs, Stach et al. in [22] applied a scalable divide and conquer strategy to speed up RCGA. In divide and conquer RCGA, the whole training data are divided into several subsets. Then, a FCM model can be learned from each subset by RCGA. And these learned FCM models are averaged as the final solution.

Particle swarm optimization (PSO) was also extended to solve the optimization problem of training FCM by Parsopoulos et al. in [23]. The learning algorithm aims to reduce the searching space continually and find a small region of candidate FCM models which is close to the real FCM model.

In addition of the above learning algorithms, other population-based algorithms were also used to learning FCMs. For example, a simulated annealing (SA) based FCM learning algorithm was proposed by Alizadeh et al. in [24]. In the learning algorithm, the optimal object is the same as the one in [21], except that the optimization algorithm is changed to SA. Chen et al. [25] proposed an ant colony optimization (ACO) algorithm for training FCMs with no more than 40 nodes, where the weights were discretized. Additionally, FCMs were also applied to solve many practical problems. In a recent work by Chen et al., biological GRNs were modeled as FCMs, and the author proposed a hybrid method which combines inherently continuous ACO algorithm with a decomposition-based optimization strategy to reconstruct biological GRNs with 100 genes from gene expression data [6].

Acampora et al. [26] introduced a memetic algorithm to generate FCM models from historical data without a priori knowledge. Extensive comparative studies performed on both synthetic and real-world data verified the competitive performance of the memetic algorithm.

However, FCMs learned by most existing algorithms are always small-scale with dozens of nodes, and only the ACO_RD_ proposed in [6] used FCMs to reconstruct gene regulatory networks (GRNs) with 100 nodes. Thus, there is a demand to develop methods capable of training large-scale FCMs based on time series data.

In our previous work, multi-agent systems and GAs are combined to form a new algorithm named as a multi-agent genetic algorithm (MAGA) for large-scale global numerical optimization [27]. The results shown MAGA performed well even for the optimization problems with 10 000 decision variables. In [28, 29], MAGA was also used as the learning algorithm to solve constraint satisfaction problems and combinatorial optimization problems, and the experiment results show a good performance. MAGA was extended to successfully solve the big optimization problem extracted from EEG data analysis mentioned above [30, 31]. Moreover, in [32], a new version of MAGA, termed as dynamic MAGA (dMAGA), was proposed, which can effectively train FCMs with 200 nodes (40 000 variables). However, dMAGA formulated the FCM training problem as one single optimization problem, where all weights are determined simultaneously. Such formulation prevents dMAGA from being able to efficiently handle even larger FCMs, such as FCMs with 500 nodes.

In fact, to learn an FCM can be considered as to learn how each node in the FCM is affected by other nodes. Thus, in order to make a training algorithm for FCMs scalable to a large number of nodes, we can decompose an FCM learning problem into multiple optimization problems, where each optimization problem corresponds to the training of a single node. That is to say, for training an FCM with *N* nodes, the original optimization problem having *N* × *N* weights (decision variables), will be decomposed into *N* optimization problems with each having only *N* decision variables. A similar idea has also been reported in [6, 33] in reconstructing GRNs based on FCMs, which successfully reduces the size of the optimization algorithm and favors a distributed implementation. Nevertheless, efficiently solving *N* optimization problems with *N* decision variables remains to be challenging, in particular when *N* becomes large.

To take full advantages of both dMAGA and the above-mentioned decomposition-based optimization approach, this work proposes a new algorithm for training larger FCMs by combining the decomposition based approach with dMAGA, which is termed as dMAGA-FCM_D_. FCMs with various sizes are used to verify the performance of the proposed dMAGA-FCM_D_. The results show that dMAGA-FCM_D_ is able to effectively train FCMs with 500 nodes and significantly enhance the performance of the original dMAGA. Moreover, dMAGA-FCM_D_ is employed to reconstruct a biological GRN, which is a challenging real-world problem. The proposed dMAGA-FCM_D_ is shown to outperform a few state-of-the-art algorithms on the benchmark problem DREAM4 [34], which the *Data Error* is as low as 0.2, where the results of other algorithms are around 0.4. DREAM4 project is a GRNs inference challenge which aims at reconstructing network structure from simulated steady-state and time series data.

## Methods

### Decomposition-based FCM for GRNs reconstruction

The relations between different concepts in a complex system can be described as a directed graph which consists of nodes and weighted edges. To adequately describe the concepts, it is necessary to use real number to quantity the expression level of each concept. For an FCM consists of *N* concept nodes, the state values of these nodes are described as a vector ***C***:

1 $$ C=\left[{C}_1,{C}_2, \dots, {C}_N\right] $$

where, *C*_*i*_∈[0, 1], *i* = (1, 2, …, *N*). Once the state value of each node is described, we need to describe the relations between nodes. Here, we use a an *N* × *N* weight matrix ***w*** to define the relations, which is also the candidate solution to the FCM learning problem,

2 $$ \boldsymbol{w}=\left[\begin{array}{cccc}\hfill {w}_{11}\hfill & \hfill {w}_{12}\hfill & \hfill \dots \hfill & \hfill {w}_{1 N}\hfill \\ {}\hfill {w}_{21}\hfill & \hfill {w}_{22}\hfill & \hfill \dots \hfill & \hfill {w}_{2 N}\hfill \\ {}\hfill \vdots \hfill & \hfill \vdots \hfill & \hfill \ddots \hfill & \hfill \vdots \hfill \\ {}\hfill {w}_{N1}\hfill & \hfill {w}_{N2}\hfill & \hfill \dots \hfill & \hfill {w}_{N N}\hfill \end{array}\right] $$

**Fig. 1:**
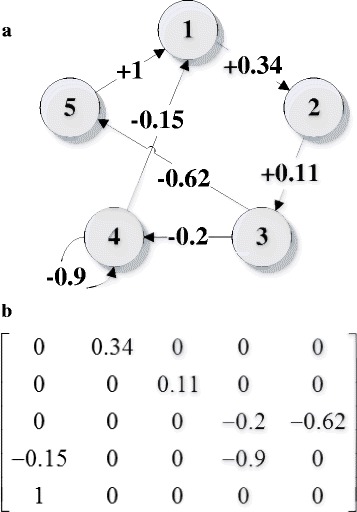
A simple example of FCMs (**a**) FCM structure, (**b**) the weight matrix

A weight is assigned to an edge connecting any two nodes to quantify the strength and the type of the relations between the two nodes. The absolute value of a weight represents how strong the source node affects the target nodes, while the positive or negative sign of weights denotes an excitatory or inhibitory relation [25]. In (2), all weights *w*_*ij*_ are in the range of [-1, 1], representing the causal relations between nodes *i* and *j*, *i*, *j* = 1, 2, …, *N*. Fig. 1a shows a simple FCM with 5 nodes and 7 edges, and the corresponding weight matrix is presented in Fig. 1b, where, e.g., *w*_12_ = +0.34 indicates that there is an excitatory edge pointing from node 1 to node 2 with a strength of 0.34. *w*_54_ = 0 means there is no causal relation between nodes 5 and 4. Similarly, *w*_44_ = -0.9 suggests that node 4 has a negative effect of feedback regulatory on itself. The higher the absolute values of the weights is, the stronger the relations.

**Fig. 2:**
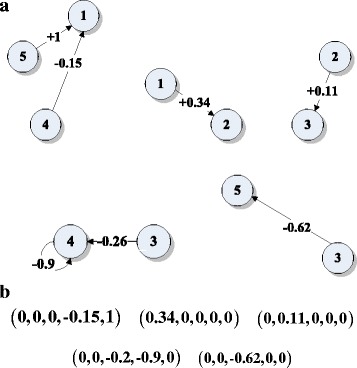
The five single sub-maps for the example FCM in Fig. 1 (**a**) 5 single sub-maps, (**b**) the corresponding weight relations

In fact, an FCM with *N* nodes can be decomposed into *N* single sub-maps in the following way. For simplicity, we focus on the input information of each node. Each node and its input nodes can be regarded as a sub-map. Every single map corresponds to a column vector in the weight matrix. In other words, the weight matrix in (2) can be represented as ***w*** = [***w***_1_, ***w***_2_, …, ***w***_*N*_], where *w*_*i*_ = (*w*_*i*1_, *w*_*i*2_, …, *w*_*iN*_), *i* = 1, 2, …, *N*. For example, the FCM in Fig. 1 can be decomposed into 5 sub-maps shown in Fig. 2a and b shows the corresponding weight relations of each single sub-map.

When the activation degree of nodes are produced, we use the following equation to predict the activation degree *C*_*i*_^*t* + 1^ based on the known values *C*_*i*_^*t*^,

3 $$ {C}_i^{t+1}= g\left({\displaystyle \sum_{j=1}^N{w}_{j i}{C}_j^t}\right),\forall i\in \left\{1,\ 2, \dots,\ N\right\} $$

where *C*_*i*_^*t*^ is the state value node *i* at the *t-*th iteration in one response, and *g*(⋅) is a sigmoid activation function that maps the activation level to the unit interval [0, 1],

4 $$ g(x)=\frac{1}{1+{e}^{-\lambda x}} $$

where parameter *λ* decides the slop of the function, and a small value lead to highly nonlinear model. Logistic transformation function is the most commonly used function in FCMs and offers significantly greater advantages than other functions [35, 36]. According to the recommendation in [37], we set 5 as the value of *λ* in this paper.

FCM learning algorithms aims to find the relations between different concepts from response sequences. In computation terms, the objective is to learn the best interconnection matrix which performs the best on simulating the response sequences. Specifically, the error between the responses generated by candidate FCM and observed response sequences are formulated as an optimization expression, and learning algorithms are used to minimize it. And the error mentioned above is labeled as *Data_Error*,

5 $$ Data\_ Error\left(\boldsymbol{w}\right)=\frac{1}{N{N}_s\left({N}_t-1\right)}{\displaystyle \sum_{n=1}^N{\displaystyle \sum_{s=1}^{N_s}{\displaystyle \sum_{t=1}^{N_t-1}{\left({C}_n^t(s)-{\widehat{C}}_n^t(s)\right)}^2}}} $$

where *N*_*s*_ is the number of response sequences, *N*_*t*_ is the number of time instants in the response sequences, *C*_*n*_^*t*^(*s*) is the *t-*th desired state value of node *n* in the *s-*th response sequence, and *Ĉ*_*n*_^*t*^(*s*) is the *t-*th state value of node *n* in the *s-*th generated response sequence.

From the decomposition point of view, *Data_Error* in (5) is actually averaged over the data error of each node and can be re-formulated as

6 $$ Data\_ Erro r\left(\boldsymbol{w}\right)=\frac{1}{N}{\displaystyle \sum_{n=1}^N\left(\frac{1}{N_s\left({N}_t-1\right)}{\displaystyle \sum_{s=1}^{N_s}{\displaystyle \sum_{t=1}^{N_t-1}{\left({C}_n^t(s)-{\widehat{C}}_n^t(s)\right)}^2}}\right)=}\frac{1}{N}{\displaystyle \sum_{n=1}^N Data\_ Erro{r}_n\left({\boldsymbol{w}}_n\right)} $$

Thus, the data error of node *i* can be represented as follows,

7 $$ Data\_ Erro{r}_i\left({\boldsymbol{w}}_i\right)=\frac{1}{N_s\left({N}_t-1\right)}{\displaystyle \sum_{s=1}^{N_s}{\displaystyle \sum_{t=1}^{N_t-1}{\left({C}_i^t(s)-{\widehat{C}}_i^t(s)\right)}^2}} $$

When we calculate *Ĉ*_*i*_^*t*^(*s*), the state values of input nodes to node *i* in the desired response sequences are used. In this way, the data error of each node can be calculated independently. In the following text, Eq. (7) is used as the objective function for optimizing the weights of the *i-*th sub-map.

### Decomposition-based dMAGA for training FCMs

Different from the dMAGA proposed in [32], which is used to optimize the whole weight matrix simultaneously, dMAGA-FCM_D_ proposed in this paper optimizes the column vectors of the weight matrix independently. In the following, we first define the agents used in dMAGA, and then introduce the genetic operators to be performed on the agents. Finally, the detailed implementation of dMAGA-FCM_D_ is provided.

### Definition of the agents

In dMAGA-FCM_D_, each candidate weight column vector ***w***_*i*_ is regarded as an agent, where *i* means the *i-*th single sub-map.

#### Definition 1

For a FCM with *N* nodes, each agent is represented as an *N*-dimensional vector. Once the representation of agent is determined, the most important thing is to define the expression of energy of each agent. In this work, the energy is the negative value of *Data_Error* defined in (7). The dMAGA-FCM_D_ aims to increase the energy of each agent as much as possible in order to survive in the environment, which is equivalent to decrease the *Data_Error*. To realize the local perceptivity of the agents, the environment is organized in a lattice-like structure, in which each agent competes or cooperates with their neighbors. An agent interacts with its neighbors, exchanging information with each other, thereby achieving global information share. The lattice in which all agents live is defined as follows.

#### Definition 2

All agents live in a lattice-like environment, ***L***, which is called an agent lattice. The size of ***L*** is ***L***_size_ × ***L***_size_, where ***L***_size_ is an integer. Each agent is fixed on a lattice-point and can only interact with its neighbors. The agent located at (*a*, *b*) is represented as ***L***_*a*,*b*_, *a*, *b* = 1, 2, …, ***L***_size_, then the neighbors of ***L***_*a*,*b*_, ***Neighbors***_*a*,*b*_, are defined as follows.

8 $$ \boldsymbol{Neighbor}{{\boldsymbol{s}}_a}_{, b}\subseteq \left\{{{\boldsymbol{L}}_{a\mathit{\hbox{'}}}}_{, b},{\boldsymbol{L}}_a{{}_{, b}}_{\mathit{\hbox{'}}},{\boldsymbol{L}}_a{{}_{, b}}_{"},{{\boldsymbol{L}}_a}_{", b}\right\} $$

where $$ {a}^{\prime }=\left\{\begin{array}{r} a-1,\  a\ne 1\\ {}{\boldsymbol{L}}_{size},\  a=1\end{array}\right.,\kern0.5em {b}^{\prime }=\left\{\begin{array}{r} b-1,\  b\ne 1\\ {}{\boldsymbol{L}}_{size},\  b=1\end{array}\right.,\kern0.5em {a}^{{\prime\prime} }=\left\{\begin{array}{l} a+1,\  a\ne {\boldsymbol{L}}_{size}\\ {}\kern1.62em 1,\  a={\boldsymbol{L}}_{size}\end{array}\right.,\kern0.5em \mathrm{and}\kern0.5em {b}^{{\prime\prime} }=\left\{\begin{array}{r} b+1,\  b\ne {\boldsymbol{L}}_{size}\\ {}1,\  b={\boldsymbol{L}}_{size}\end{array}\right.. $$

**Fig. 3:**
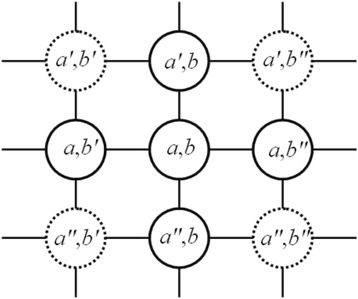
The topology of the defined agent neighborhood

As shown in Fig. 3, there are four agents at most in the neighborhood for each agent. In the standard MAGA, each agent has to compete with the other four agents around it, in other words, the four agents around it are all its neighbors, so the agents with low energy are eliminated early, which make MAGA lost the diversity of the population under the huge selection pressure. Thus, in dMAGA-FCM_D_, in order to tune the selection pressure, each agent can select its neighbors dynamically according to the energy. Namely, there is no need to compete with all the agents around it. Thus, ***Neighbors***_*a,b*_ in is a subset of (8).

To dynamically determine the neighbor of each agent, at the beginning of each generation, all agents will be sorted according to a decreasing order of their energy and evenly divided into four levels. And the agents in the first/second/third/fourth level can select four/three/two/one neighbor(s) randomly from (8). In this way, an agent with a lower level of energy will have a smaller number of neighbors, thereby improving its chance to survive than those in MAGA.

### Genetic operators for agents

Four genetic operators are used to guide the evolutionary search, which are the neighborhood competition, the crossover, the mutation, and the self-learning operator, of which the neighborhood competition and crossover operators account for competition and cooperator among agents, while the mutation and self-learning operators are dedicated to improving the agents’ ability of local search and making use of knowledge to survive. Suppose the four operators are performed on agent *L*_*a*,*b*_ = (*l*_1_, *l*_2_, …, *l*_*N*_), and **Max**_*a*,*b*_ = (*m*_1_, *m*_2_, …, *m*_*N*_) is the agent with the maximum energy in neighbors of ***L***_*a*,*b*_.

#### Neighborhood competition operator

If ***L***_*a*,*b*_ satisfies **Energy**(***L***_*a*,*b*_) > **Energy**(**Max**_*a*,*b*_), which means ***L***_*a*,*b*_ defeats all the neighbors and keep the current solution in lattice; otherwise, ***L***_*a*,*b*_ lost the change to survive and have to be replaced by **Max**_*a*,*b*_ which is the energy with maximum energy in the neighborhood. There are also two strategies determine how the agent ***L***_*a*,*b*_ would be replaced. That is, if *U*(0, 1) < *P*_*o*_, strategy 1 is adopted; otherwise, strategy 2 is used. Here, *P*_*o*_ is a predefined probability, and *U*(0, 1) is a random number uniformly distributed in the interval of [0, 1].

#### Strategy 1

A new agent **New**_*a*,*b*_ = (*e*_1_, *e*_2_, …, *e*_*N*_) is generated as follows:

9 $$ {e}_i=\left\{\begin{array}{l}\hbox{-} 1,\kern0.75em \omega <\hbox{-} 1\\ {}\ 1,\kern0.75em \omega >1\\ {}\omega, \kern0.75em \mathrm{otherwise}\end{array}\right.\kern1em ,\kern0.5em \mathrm{where}\kern1.5em \omega ={m}_i+ U\left(-1,\ 1\right)\times \left({m}_i-{l}_i\right),\kern0.5em  i=1,\ 2, \dots, N $$

Since there might be still some useful information in ***L***_*a*,*b*_ even if it is a loser, Strategy 1 uses both ***L***_*a*,*b*_ and **Max**_*a*,*b*_ to generate a new agent, which is used to replace ***L***_*a*,*b*_.

#### Strategy 2

Randomly select two integers *k* and *s* satisfying 1 *< k* < *s* < *N*, then a new agent is generated as follows to replace ***L***_*a*,*b*_,

10 $$ \mathbf{N}\mathbf{e}{{\mathbf{w}}_a}_{, b}=\left({m}_1,{m}_2, \dots, {m_k}_{-1},{m}_s,{m_s}_{-1}, \dots, {m_k}_{+1},{m}_k,{m_s}_{+1}, \dots, {m}_N\right) $$

#### Crossover and mutation operators

The orthogonal crossover and Gaussian mutation operators are applied on ***L***_*a,b*_ and **Max**_*a,b*_. The reader are referred to [32] for the details of these operators.

#### Self-learning operator

In MAGA, a small scale MAGA is introduced as the self-learning operator for agents to be able to use knowledge, which however, is still a sort of random-based local search strategy. Therefore, to make use of the properties of FCMs, in dMAGA, a one dimensional search strategy is adopted to implement the self-learning operator. In this work, we also perform this self-learning operator on ***L***_*a*,*b*_, and more details can be found in [32].

### Implementation of dMAGA-FCM_D_

dMAGA-FCM_D_ optimizes each sub-map sequentially. For each sub-map, the neighbors of each agent is first determined, then each agent compete with its neighbors according to their energy, and the agent with a higher level of energy survives in the population. Once the competition is performed, the crossover and mutation operators are performed on each agent with a probability *P*_*c*_ and *P*_*m*_, respectively. Then, the best agent in the current generation improves its energy through self-learning operator.

Algorithm 1 shows more details of dMAGA-FCM_D_, where ***L***_*i*_^*t*^ represents the agent lattice at the *t-*th generation for the *i-*th single sub-map, *CBest*_*i*_^*t*^ represents the best agent in the *t-*th generation for the *i-*th single sub-map, and *Best*_*i*_ represents the best agent found for the *i-*th single sub-map.

## Results

### Experiments on synthetic FCMs

In this section, the dMAGA-FCM_D_ is tested on synthetic FCMs. For fully test the performance of the dMAGA-FCM_D_, the scale of FCMs is varying from 5 to 500 nodes. In order to show the improvement of dMAGA-FCM_D_ over dMAGA, extensive comparisons are made between these two algorithms. In addition, dMAGA-FCM_D_ is compared with three representative existing methods based on evolutionary algorithms, namely, RCGA [21], ACO_RD_ (an improved variant of ACO) [6] and differential evolution (DE) [38], where the results of RCGA and DE are taken from the literature. ACO_RD_ and dMAGA are run under the same experimental settings as those of dMAGA-FCM_D_. All experiments on dMAGA-FCM_D_ are conducted for the same parameter settings, which are given in [32].

*Data_Error*, which is presented in (5), is an important measure to evaluate the performance of different learning algorithms. Unlike the *Data_Error* is used to evaluate the fitting ability of time sequences, *Model_Error* is a direct comparison between the weights of the learned model and the target model,

11 $$ Model\_ Error=\frac{1}{N^2}{\displaystyle \sum_{i=1}^N{\displaystyle \sum_{j=1}^N\left|{w}_{i j}-{\widehat{w}}_{i j}\right|}} $$

where *ŵ*_*ij*_ is the learned weight of between nodes *i* to *j*.

In order to evaluate the performance of the algorithms under comparison in predicting the existence of edges, the problem of training FCMs is extended and transformed into a binary classification problem. That is, the target FCM model and the learned FCM model are transformed into binary networks according to a predefined threshold. The absolute weights that are larger than the threshold are transformed to 1; otherwise 0. Once the transformational rule is determined, we need to choose a value of the predefined threshold. In this paper, we choose 0.05 as the value of the threshold which is the same value used in [37]. In FCMs, we usually think that the causal relation with strength less than 0.05 usually has no significance in practical problems.

The author of [37] also gives the definition of positive and negative edges. According to the definition, the edges with absolute weights larger than 0.05 are identified as negative edges; otherwise, they are identified as positive edges. The *SS mean* is employed to evaluate the performance,

12 $$ S S\kern0.5em  mean=\frac{2\times S ensitivity\times S pecificity}{Sensitivity+ Specificity} $$

where

13 $$ Sensitivity=\frac{N_{TP}}{N_{TP}+{N}_{FN}} $$

14 $$ Specificity=\frac{N_{TN}}{N_{TN}+{N}_{FP}} $$

where *TP*, *FN*, *TN*, and *FP* are defined in Table 1. *T* (true) means the correctly identified edges. *F* (false) means the edge is identified as the opposite character. *P* (positive) means positive edge and *N* (negative) means negative edge which has defined above (12). For example, *N*_*FP*_ is the number of false positive edges, which means the number of negative edges that are incorrectly identified as positive edges.

**Table 1:** Definition of *TP*, *FN*, *TN*, and *FP*

		Target networks	
		0	1
Learned networks	0	*TP*	*FP*
	1	*FN*	*TN*

In this paper, the artificial response time sequences used to train FCM models are generated by a two-step method proposed in [25]. First, random real numbers which should be within the interval [-1, 1] are assigned to the weights of the interconnection matrix, an additional file shows this in more detail (see Additional file 1). However, according to the viewpoint in [25], which is if the weight between two nodes is smaller than 0.05, the relation between these two nodes can be ignored in practical application, we check each weight whether its absolute value is smaller than 0.05. If so, the weight will be set to 0. Second, the state value of response time sequences at the initial time point are assigned by random value ranging from 0 to 1. Then, the subsequent time sequences can be generated according to (3) based on the FCM model and initial state values.

**Fig. 4:**
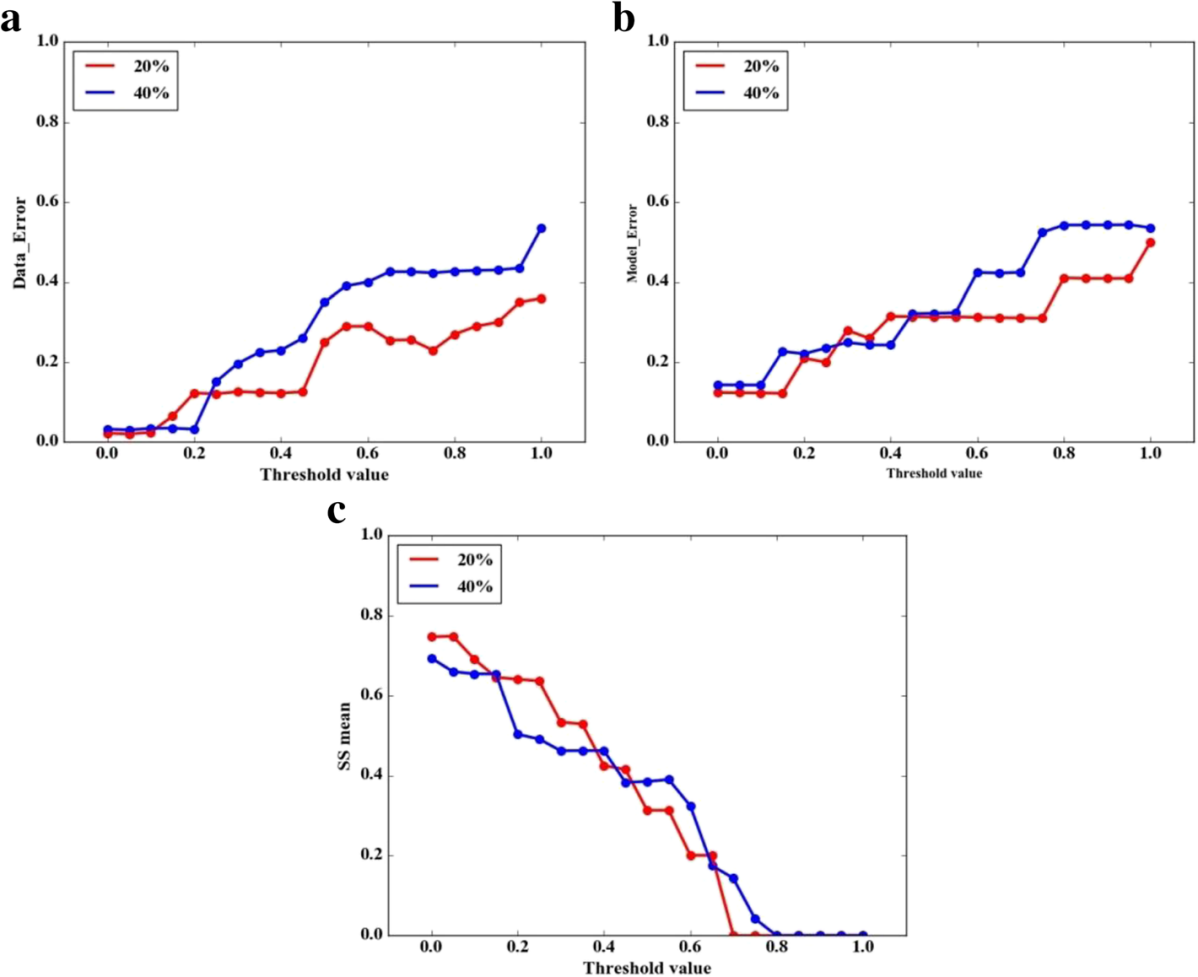
Comparison in terms of (**a**) *Data_Error*, (**b**) *Model_Error* and (**c**) *SS mean* on FCMs (10 nodes) with various of threshold value which ranges from 0 to 1. The red line and blue line show the comparison experiments for FCMs with 20% density and 40% density, respectively

Here, the threshold value (0.05) used in this work is always a default parameter in many work [6, 12, 25] about FCMs. However, different threshold value will affect the algorithm performance. In order to explore the impact of the threshold value on algorithm performance, we conduct the following experiment. When the network (10 Node, 20% density and 40% density) was learned, we set the value of threshold ranges from 0 to 1 in the step of 0.05 and get a series of networks. Then we calculate the *Data_Error*, *Model_Error* and *SS mean* for networks with different threshold values. From Fig. 4, we can see that the value of threshold affect the algorithm performance greatly, no matter what the density of the network, the performance of the algorithm decreases with the increase of threshold value. When the threshold value is bigger than 0.6, the value of *SS mean* is even 0 which means there is no existence of edge in this network is predicted correctly. Thus, it is important to select an appropriate threshold for the performance of the algorithm.

In real-world, the network structures of FCMs are always sparse. There are only a fraction of links between nodes in FCMs. For this reason, we need to control the density of the random FCM model. When we generate the FCM models by the method mentioned above, a large part of weights are assigned to 0 and the others are set to nonzero random real numbers. For example, if the edge density of 20% is expected for an FCM with 10 nodes, 20 random edges (there are 10 × 10 = 100 edges with the full connected graph, then 20% of 100 edges is 20) will be selected and random values will be assigned to these edges.

**Fig. 5:**
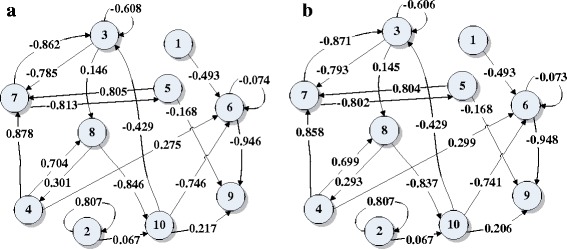
**a** The original FCM with 10 nodes, **b** the learned FCM by dMAGA-FCM_D_

**Fig. 6:**
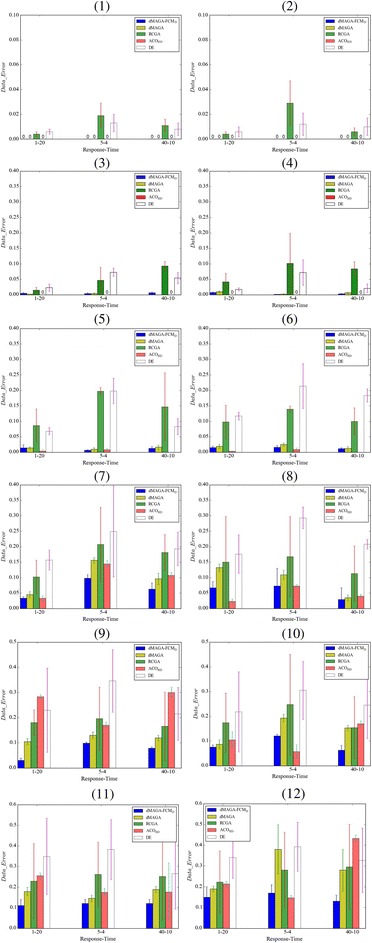
Comparison in terms of *Data_Error* on FCMs with various number of nodes. (*1*) 5 nodes, density = 20%. (*2*) 5 nodes, density = 40%. (*3*) 10 nodes, density = 20%. (*4*) 10 nodes, density = 40%. (*5*) 20 nodes, density = 20%. (*6*) 20 nodes, density = 40%. (*7*) 40 nodes, density = 20%. (*8*) 40 nodes, density = 40%. (*9*) 100 nodes, density = 20%. (*10*) 100 nodes, density = 40%. (11) 200 nodes, density = 20%. (12) 200 nodes, Density = 40%

**Fig. 7:**
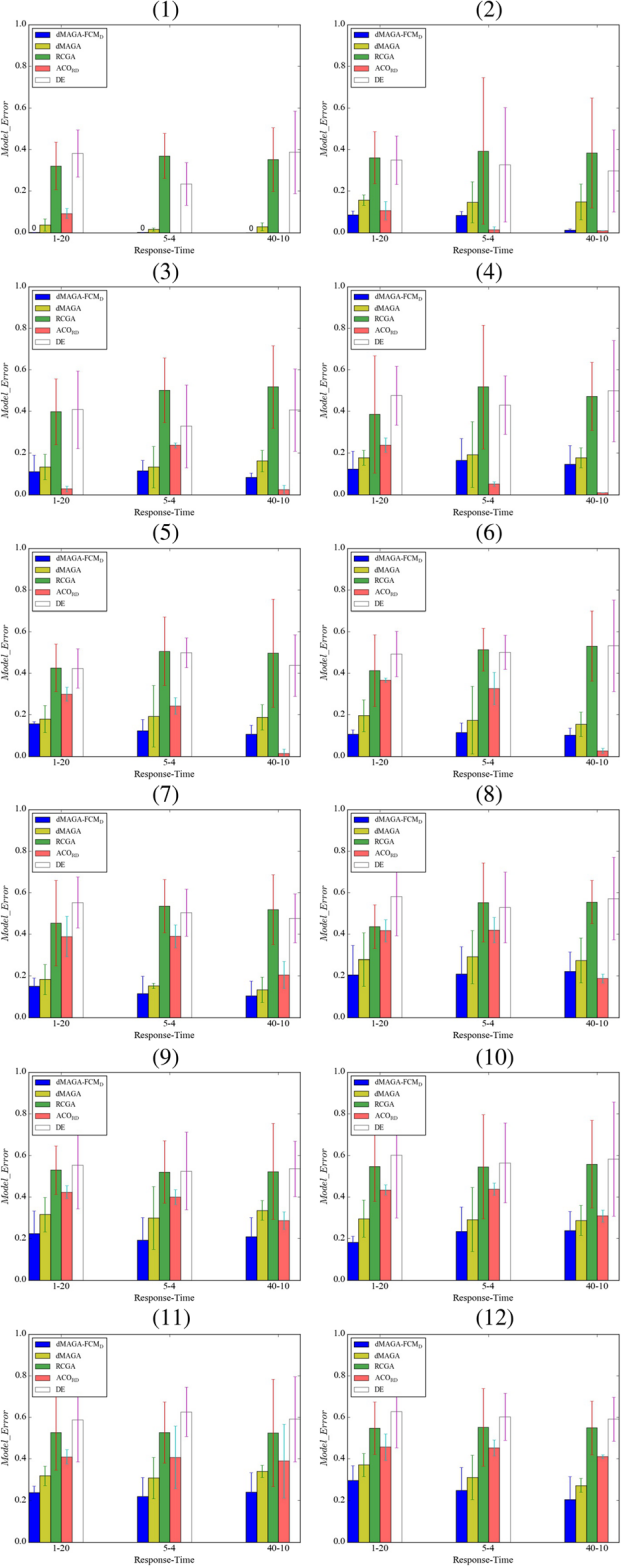
Comparison in terms of *Model_Error* on FCMs with various number of nodes. (*1*) 5 nodes, density = 20%. (*2*) 5 nodes, density = 40%. (*3*) 10 nodes, density = 20%. (*4*) 10 nodes, density = 40%. (*5*) 20 nodes, density = 20%. (*6*) 20 nodes, density = 40%. (*7*) 40 nodes, density = 20%. (*8*) 40 nodes, density = 40%. (*9*) 100 nodes, density = 20%. (*10*) 100 nodes, density = 40%. (*11*) 200 nodes, density = 20%. (*12*) 200 nodes, density = 40%

The size of generated FCM varies from 5 to 500 (*N* = 5, 10, 20, 40, 100, 200, 300, and 500), and for each size, the edge density of 20% and 40% are used. In order to compare with other algorithms, experiments on three scenarios are conducted. The first scenario has one response sequence with 20 time points each (*N*_*s*_ = 1, *N*_*t*_ = 20), the second scenario has five response sequences with four time points each (*N*_*s*_ = 5, *N*_*t*_ = 4), and the third scenario has 40 response sequences with 10 time points each (*N*_*s*_ = 40, *N*_*t*_ = 10). Figure 5a shows an original FCM with 10 nodes with 20% edge density and Fig. 5b shows the corresponding FCM learned by dMAGA-FCM_D_. By comparing the original and learned FCMs, we see that the network structure is fully correctly learned and the errors between the original and learned weights are smaller than 0.02. Comprehensive comparative results in terms of *Data_Error* on FCMs with 5 ~ 200 nodes are presented in Fig. 6, and a detailed comparison of the FCMs with 300 and 500 nodes is reported in Table 2. The comparison in terms of *Model_Error* is given in Fig. 7 and Table 3. The results are averaged over 30 independent runs for FCMs with 5 ~ 200 nodes and 10 independent runs for FCMs with 300 and 500 nodes.

**Table 2:** Comparison in terms of *Data Error* on larger synthetic FCMs (Average ± Standard deviation)

#Nodes	Edge density	*N*_s_-*N*_*t*_	dMAGA-FCM_D_	dMAGA
300	20%	1–20	0.105 ± 0.021	0.297 ± 0.102
		5–4	0.125 ± 0.053	0.247 ± 0.031
		40–10	0.118 ± 0.037	0.336 ± 0.025
	40%	1–20	0.121 ± 0.082	0.214 ± 0.112
		5–4	0.063 ± 0.019	0.351 ± 0.051
		40–10	0.139 ± 0.070	0.298 ± 0.042
500	20%	1–20	0.145 ± 0.107	0.368 ± 0.114
		5–4	0.186 ± 0.034	0.420 ± 0.027
		40–10	0.156 ± 0.072	0.395 ± 0.064
	40%	1–20	0.130 ± 0.056	0.348 ± 0.108
		5–4	0.164 ± 0.081	0.416 ± 0.086
		40–10	0.173 ± 0.022	0.404 ± 0.053

**Table 3:** Comparison in terms of *Model_Error* on larger synthetic FCMs (Average ± Standard deviation)

#Nodes	Edge density	*N*_*s*_-*N*_*t*_	dMAGA-FCM_D_	dMAGA
300	20%	1–20	0.305 ± 0.024	0.371 ± 0.062
		5–4	0.321 ± 0.072	0.382 ± 0.028
		40–10	0.241 ± 0.010	0.298 ± 0.091
	40%	1–20	0.352 ± 0.016	0.386 ± 0.083
		5–4	0.257 ± 0.051	0.296 ± 0.024
		40–10	0.310 ± 0.027	0.390 ± 0.109
500	20%	1–20	0.323 ± 0.032	0.401 ± 0.102
		5–4	0.317 ± 0.017	0.414 ± 0.023
		40–10	0.321 ± 0.045	0.374 ± 0.127
	40%	1–20	0.285 ± 0.081	0.339 ± 0.134
		5–4	0.379 ± 0.015	0.430 ± 0.085
		40–10	0.392 ± 0.029	0.407 ± 0.141

As can be seen, in terms of *Data_Error*, dMAGA-FCM_D_, dMAGA and ACO_RD_ all reach 0.000 for FCMs with 5 nodes, no matter whether the edge density is 20% or 40%. For FCMs with 10 and 20 nodes, although *Data_Error* of dMAGA-FCM_D_ is larger than that of ACO_RD_, it is much smaller than those of RCGA and DE. For FCMs with 40 ~ 200 nodes, dMAGA-FCM_D_ outperforms ACO_RD_ on 13 out of 16 FCMs and outperforms all other methods with different edge densities and *N*_*s*_-*N*_*t*_ combinations. Moreover, Table 2 shows that dMAGA-FCM_D_ clearly outperforms dMAGA on FCMs with 300 and 500 nodes.

The comparison in terms of *Model_Error* shows that for FCMs with 5 ~ 200 nodes, dMAGA-FCM_D_ always outperforms dMAGA, RCGA, and DE. For FCMs with 10 nodes, ACO_RD_ performs better than dMAGA-FCM_D_, but for FCMs with 20 and 40 nodes, dMAGA-FCM_D_ outperforms ACO_RD_ on 9 out of all the 12 cases regardless the edge density and *N*_*s*_-*N*_*t*_ combination. Moreover, all the averaged *Model_Error* of dMAGA-FCM_D_ for FCMs with 300 and 500 nodes is smaller than 0.4,which is consistently smaller than that of dMAGA.

In addition, the above results show that the performance of dMAGA-FCM_D_ in terms of *Data_Error* and *Model_Error* is not very sensitive to the number of nodes, the number of response sequences, and the number of time points in a certain range, where at least four time-points are required and the number of nodes are limited less than 500. It indicates that dMAGA-FCM_D_ is robust and is scalable to the size of FCMs to a certain extent.

**Fig. 8:**
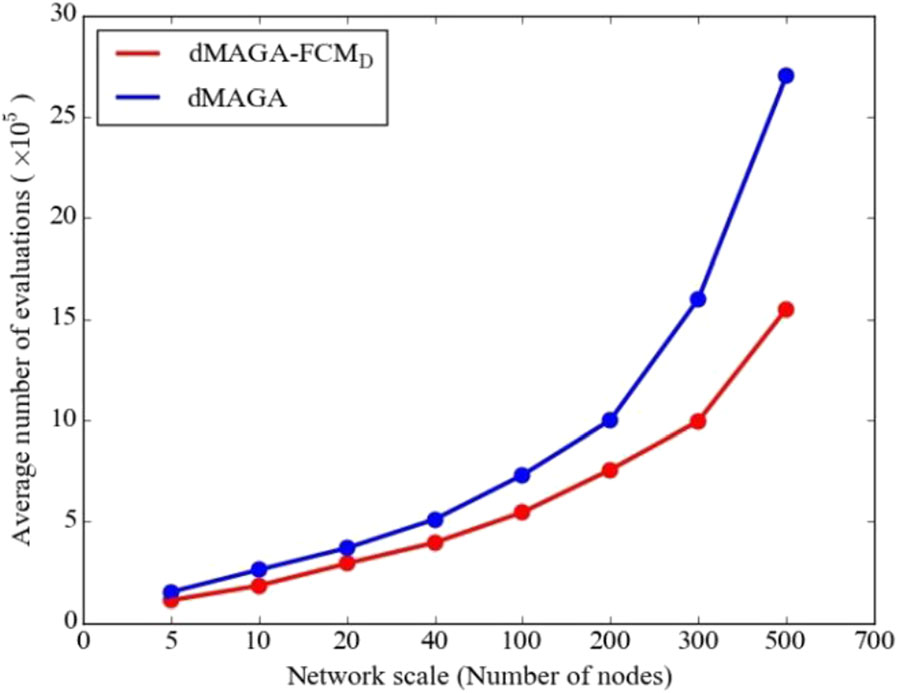
The number of fitness evaluations consumed by dMAGA-FCM_D_ and dMAGA, respectively

Note also that, the experimental results of dMAGA-FCM_D_ are obtained with less than 1.5 × 10^6^ fitness function evaluations (see Fig. 8), whereas the results of compared algorithms use 3 × 10^6^ fitness function evaluations, which are much larger than that of dMAGA-FCM_D_. As can be seen from Fig. 8, dMAGA-FCM_D_ efficiently reduces the computational cost compared with dMAGA, especially for large-scale FCMs. Therefore, dMAGA-FCM_D_ has exhibited better performance with lower computational cost compared to the state-of-the-art, demonstrating that the algorithm is very competitive for solving large-scale problems.

**Fig. 9:**
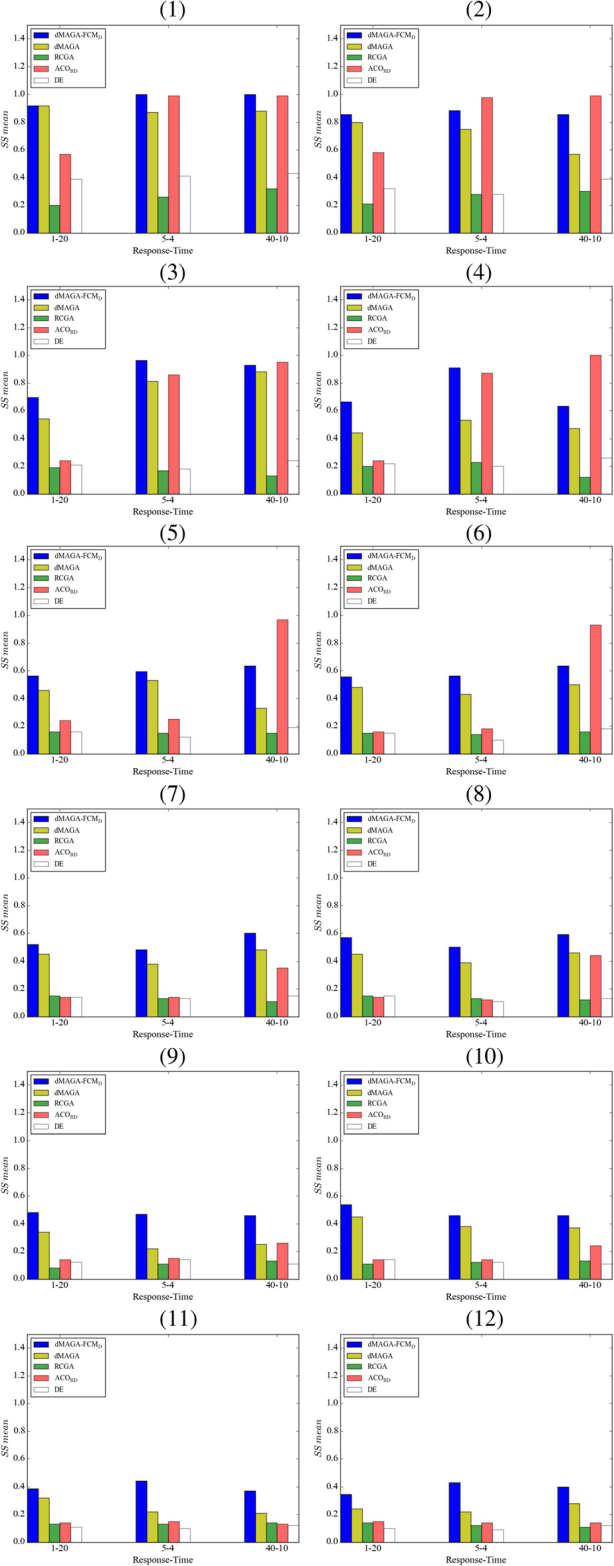
Comparison in terms of *SS Mean* on FCMs with various number of nodes. (*1*) 5 nodes Density = 20%, (*2*) 5 nodes Density = 40%, (*3*) 10 nodes, density = 20%, (*4*) 10 nodes, density = 40%, (*5*) 20 nodes, density = 20%, (*6*) 20 nodes, density = 40%, (*7*) 40 nodes, density = 20%, (*8*) 40 nodes, density = 40%, (*9*) 100 nodes, density = 20%, (*10*) 100 nodes, density = 40%, (*11*) 200 nodes, density = 20%, (*12*) 200 nodes, density = 40%

Figure 9 report the performance of dMAGA-FCM_D_ in terms of *SS Mean*. As we can see, for FCMs with 5 ~ 20 nodes, dMAGA-FCM_D_ consistently outperforms dMAGA, RCGA and DE, and also outperforms ACO_RD_ on 12 out of all the 18 cases. For FCMs with 40 ~ 200 nodes, dMAGA-FCM_D_ performs better than all the other learning algorithms on all cases. Table 4 shows the comparative results in terms of *SS mean* of dMAGA-FCM_D_ and dMAGA for FCMs with 300 and 500 nodes. As can be seen, compared with dMAGA, dMAGA-FCM_D_ significantly enhances the ability of dMAGA in training larger FCMs.

**Table 4:** Comparison in terms of *SS mean* on larger synthetic FCMs

#Nodes	Edge density	*N*_*s*_-*N*_*t*_	dMAGA-FCM_D_	dMAGA
300	20%	1–20	0.329	0.309
		5–4	0.303	0.215
		40–10	0.425	0.302
	40%	1–20	0.374	0.290
		5–4	0.384	0.296
		40–10	0.455	0.388
500	20%	1–20	0.237	0.163
		5–4	0.243	0.177
		40–10	0.341	0.139
	40%	1–20	0.290	0.193
		5–4	0.246	0.186
		40–10	0.342	0.151

### Experiments on DREAM4 in silico network challenge

Gene regulatory networks (GRNs) have been widely used to model, analyze and predict the behavior of biological organisms. A GRN aims to build the relationships between a set of molecular entities and is often modeled as a network composed of nodes (representing genes, proteins or metabolites) and edges (representing molecular interactions such as protein–DNA and protein–protein interactions or rather indirect relationships between genes) [39]. In this section, we employ the proposed dMAGA-FCM_D_ to reconstruct a biological GRN based on gene expression time series data, known as DREAM4 [34], a widely used benchmark for evaluating reverse engineering methods [40]. The gene expression time series data were generated based on the network structures of *Escherichia coli* and *Saccha-romyces cerevisiae*. Time series database contains a variety of network sizes with 10 and 100 genes. Perturbation and noise on expression profiles are generated by differential equations. There are five separate networks for each type of networks.

In the DREAM4 data, under the perturbations of internal noise and measurement noise, there are five time series for each gene. Each time series contains 21 time points. The first 10 time points evaluate the response of gene networks in the presence of perturbations. The perturbations are revoked at the 11-th time instant. So the last 11 time points reflect the response of gene networks after the wild type network is restored. In this experiment, we use the last 11 time points and test the performance of dMAGA-FCM_D_ in terms of *Data_Error* and *SS mean*.

**Fig. 10:**
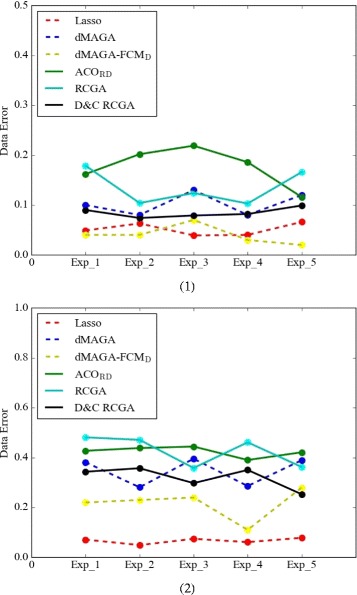
Experimental results in terms of *Data_Error* for GRNs with (*1*) 10 genes and (*2*) 100 genes. (*1*) Five experiments for GRNs with 10 genes. (*2*) Five experiments for GRNs with 100 genes

In the experiments, we perform several efficient evolutionary-based algorithms, dMAGA, RCGA, D&C RCGA and DE, one DREAM data. We also perform Lasso [41] for comparison. The experimental results are shown in Fig. 10. As can be seen, *Data_Error* of dMAGA-FCM_D_ perform the best for GRNs with 10 genes except one case (Exp_3). For GRNs with 100 genes, Lasso is better than dMAGA-FCM_D_ in terms of *Data_Error*, and dMAGA-FCM_D_ performs better than other evolutionary-based algorithms. We can also see that the decomposition strategy is always useful, because the dMAGA-FCM_D_ is always better than dMAGA, D&C RCGA, which is RCGA with decomposition strategy, is always better than RCGA no matter the number of genes is 10 or 100.

## Discussion

The proposed algorithm dMAGA-FCM_D_ enhances prediction, which is valuable for machine learning. As the decomposition strategy, dMAGA-FCM_D_ has reduced the computational cost. Reconstructed FCMs can be modeled as a big data optimization problem. Previous studies indicate that the number of variables needs to be optimized increases quadratically with the scale of FCMs, resulting an exponential increase in the number of candidate solutions. Currently, most learning algorithms can only handle small scale FCMs which number of nodes is smaller than 100. In this work, we show that the dMAGA-FCM_D_ can handle large FCMs with 300 and 500 nodes (see Table 2, 3 and 4). The experiment results also show that dMAGA-FCM_D_ efficiently reduces the computational cost compared with our previous work dMAGA, especially for large-scale FCMs, which demonstrating that the algorithm is very competitive for solving large-scale problems.

In the DREAM4 data, the dMAGA-FCM_D_ performs better than the other evolutionary-based learning algorithms. However, in the five experiments for GRNs with 100 genes, the Lasso is better than dMAGA-FCM_D._ The performed Lasso takes into account the sparseness of the network. Therefore, the properties of Lasso algorithm provide a good direction for us to improve the dMAGA-FCM_D_.

## Conclusions

In this paper, we propose a new algorithm, termed as dMAGA-FCM_D_, to train large-scale GRNs based on FCMs using time series data by introducing the decomposition based optimization approach into the dynamical multi-agent genetic algorithm. The dMAGA-FCM_D_ has shown to be well suited for learning causal relations of complex systems. Extensive experiments are conducted on both synthetic FCMs and DREAM4, a challenge to reverse GRNs from simulated time series data. Experimental results show that dMAGA-FCM_D_ is able to effectively train FCMs with up to 500 nodes and improves ability of reconstructing GRNs with high accuracy, outperforming the compared state-of-the-art learning algorithms. Our results also indicate that dMAGA-FCM_D_ is promising for solving larger-scale GRNs, which will be considered in our future work.

## Additional file

Additional file 1: Synthetic FCMs. In this work, the scale of synthetic FCMs is varying from 5 to 500 nodes, and the density is 20% and 40% for each scale. The method used to generate FCMs is as the same as the method proposed in Ref. [25], we also described the method in “Results” section, page 14. (DOCX 33 kb)

## Abbreviations

dMAGA-FCM_D_: Dynamical multi-agent genetic algorithm with the decomposition-based model
FCM: Fuzzy cognitive maps
GRN: Gene regulatory networks

## References

[1] Abbass HA. Calibrating independent component analysis with Laplacian reference for real-time EEG artifact removal. 21st International Conference on Neural Information Processing. 2014. p. 68–75.

[2] Goh SK, Abbass HA, Tan KC. Artifact removal from EEG using a multi-objective independent component analysis model. 21st International Conference on Neural Information Processing. 2014. p. 570–577.

[3] Repsilber D, Liljenström H, Andersson SGE (2002). Reverse engineering of regulatory networks: simulation studies on a genetic algorithm approach for ranking hypotheses. Biosystems.

[4] Eriksson R, Olsson B (2004). Adapting genetic regulatory models by genetic programming. Biosystems.

[5] Fomekong-Nanfack Y, Kaandorp JA, Blom J (2007). Efficient parameter estimation for spatio-temporal models of pattern formation: case study of Drosophila melanogaster. Bioinformatics.

[6] Chen Y, Mazlack LJ, Lu LJ. Inferring fuzzy cognitive map models for gene regulatory networks from gene expression data. IEEE International Conference on Bioinformatics and Biomedicine. 2012. p. 589–601

[7] Kauffman SA (1992). The origins of order: self organization and selection in evolution. J Evol Biol.

[8] Margolin AA, Wang K, Lim WK, Kustagi M, Nemenman I, Califano A (2006). Reverse engineering cellular networks. Nat Protoc.

[9] Butte AJ, Kohane IS (2000). Mutual information relevance networks: functional genomic clustering using pairwise entropy measurements. In Pac Symp Biocomput.

[10] Friedman N, Linial M, Nachman I, Pe’er D (2000). Using bayesian networks to analyze expression data. J Comput Biol.

[11] Marbach D, Costello JC, Kuffner R, Vega NM, Prill RJ, Camacho DM (2012). Wisdom of crowds for robust gene network inference. Nat Methods.

[12] Chen Y, Mazlack LJ, Ali AM, Long LJ (2015). Inferring causal networks using fuzzy cognitive maps and evolutionary algorithms with application to gene regulatory network reconstruction. Appl Soft Comput.

[13] Papageorgiou EI (2012). Learning algorithms for fuzzy cognitive maps - a review study. IEEE Trans Syst Man Cybern Part C.

[14] Kosko B (1986). Fuzzy cognitive maps. Int J Man Mach Stud.

[15] Georgopoulos VC, Malandraki GA, Stylios CD (2003). A fuzzy cognitive map approach to differential diagnosis of specific language impairment. J Artif Intel Med.

[16] Dickerson JA, Cox Z, Wurtele ES, Fulmer AW (2001). Creating metabolic and regulatory network models using fuzzy cognitive maps. North Am Fuzzy Inform Proc Conf (NAFIPS).

[17] Papageorgiou E, Groumpos P (2004). A weight adaptation method for fuzzy cognitive maps to a process control problem.

[18] Bakken BT, Gilljam M. Training to improve decision-making-system dynamics applied to higher level military operations. In: 20th International System Dynamics Conference. Palermo; 2002.

[19] Stach W, Kurgan L. Modeling software development project using fuzzy cognitive maps. Proc. 4th ASERC Workshop on Quantitative and Soft Software Engineering (QSSE’04). 2004. p. 55–60.

[20] Stach W, Kurgan L, Pedrycz W, Reformat M. Parallel fuzzy cognitive maps as a tool for modeling software development project. Proc. 2004 North American Fuzzy Information Processing Society Conf. (NAFIPS’04), Banff; 2004. p. 28–33

[21] Stac W, Kurgan L, Pedrycz W, Reformat M (2005). Genetic learning of fuzzy cognitive maps. Fuzzy Sets Syst.

[22] Stach W, Kurgan L, Pedrycz W (2010). A divide and conquer method for learning large fuzzy cognitive maps. Fuzzy Sets Syst.

[23] Papageorgiou EI, Parsopoulos KE, Stylios CS, Groumpos PP, Vrahatis MN (2005). Fuzzy cognitive maps learning using particle swarm optimization. J Intell Inf Syst.

[24] Ghazanfari M, Alizadeh S, Fathian M, Koulouriotis DE (2007). Comparing simulated annealing and genetic algorithm in learning FCM. Appl Math Comput.

[25] Chen Y, Mazlack LJ, Lu LJ. Learning fuzzy cognitive maps from data by ant colony optimization. In: Proceedings of Genetic and Evolutionary Computation Conference (GECCO). 2012. p. 9–16.

[26] Acampora G, Pedrycz W, Vitiello A (2015). A competent memetic algorithm for learning fuzzy cognitive maps. IEEE Trans Fuzzy Systems.

[27] Zhong W, Liu J, Xue M, Jiao L (2004). A multiagent genetic algorithm for global numerical optimization. IEEE Trans Syst Man Cybern Part B.

[28] Liu J, Zhong W, Jiao L (2006). A multiagent evolutionary algorithm for constraint satisfaction problems. IEEE Trans Syst Man Cybern Part B.

[29] Liu J, Zhong W, Jiao L (2010). A multiagent evolutionary algorithm for combinatorial optimization problems. IEEE Trans Syst Man Cybern Part B.

[30] Zhang Y, Zhou M, Jiang Z, Liu J. A multi-agent genetic algorithm for big optimization problems. IEEE Congr Evol Comput (CEC). 2015;703–7.

[31] Zhang Y, Liu J, Zhou M, Jiang Z (2016). A multi-objective memetic algorithm based on decomposition for big optimization problems. Memetic Comput.

[32] Liu J, Chi Y, Zhu C (2016). A dynamic multi-agent genetic algorithm for gene regulatory network reconstruction based on fuzzy cognitive maps. IEEE Trans Fuzzy Systems.

[33] Chi Y, Liu J. Reconstructing gene regulatory networks with a memetic-neural hybrid based on fuzzy cognitive maps. Natural Computing. 2016. In press.

[34] Greenfield A, Madar A, Ostrer H, Bonneau R (2010). DREAM4: combining genetic and dynamic information to identify biological networks and dynamical models. PLoS One.

[35] Aguilar J (2005). A survey about fuzzy cognitive maps papers. Int J Comput Cognition.

[36] Bueno S, Salmeron JL (2009). Benchmarking main activation functions in fuzzy cognitive maps. Expert Syst Appl.

[37] Stach W (2010). Learning and aggregation of fuzzy cognitive maps – an evolutionary approach.

[38] Papageorgiou EI, Groumpos PP (2004). Optimization of fuzzy cognitive map model in clinical radiotherapy through the differential evolution algorithm. Biomed Soft Comput Human Sci.

[39] Hecker M, Lambeck S, Toepfer S, Someren E, Guthke R (2009). Gene regulatory network inference: data integration in dynamic models-a review. Biosystems.

[40] Thomas SA, Jin Y (2014). Reconstructing gene regulatory networks: where optimization meets big data. Evol Intel.

[41] Tibshirani R (1996). Regression shrinkage and selection via the lasso. J Royal Stat Soc Series B (Methodological).

