# Supplementary material for: A time series driven decomposed evolutionary optimization approach for reconstructing large-scale gene regulatory networks based on fuzzy cognitive maps
Source: BMC Bioinformatics. 2017 May 8;18:241. doi: 10.1186/s12859-017-1657-1 (PMC5423002; doi:10.1186/s12859-017-1657-1)
Supplement: Additional file 1: — Synthetic FCMs. In this work, the scale of synthetic FCMs is varying from 5 to 500 nodes, and the density is 20% and 40% for each scale. The method used to generate FCMs is as the same as the method proposed in Ref. [25], we also described the method in “Results” section, page 14. (DOCX 33 kb) [file 12859_2017_1657_MOESM1_ESM.docx]

**Synthetic FCMs**

In this work, the scale of synthetic FCMs is varying from 5 to 500 nodes. When the number of nodes is large, it is difficult to show the specific network due to the space limitations. As in each experiment, the weight values of the FCMs were randomly generated, it is more import to give the FCMs generated method. The method used to generate FCMs is as the same as the method proposed in [25], we also described the method below Table 2. Here we show a few synthetic FCMs examples with 5 and 10 nodes.

$\left[ \begin{matrix} 0.21 & 0 & 0 & -0.57 & -0.42 \\ 0 & 0 & 0 & 0 & 0 \\ 0 & 0 & 0.73 & 0 & 0 \\ 0 & 0 & 0 & 0 & 0 \\ 0 & 0 & 0 & 0.18 & 0 \end{matrix} \right]$ $\left[ \begin{matrix} 0 & 0 & 0 & 0.7 & 0 \\ 0.08 & 0.64 & 0 & 0 & 0 \\ 0 & 0 & -0.38 & 0 & 0 \\ -0.94 & -0.14 & 0.11 & 0.66 & 0 \\ 0.32 & 0 & 0 & 0 & -0.62 \end{matrix} \right]$

(a) 5 nodes, density=20% (b) 100 nodes, density=40%

$$\left[ \begin{matrix} 0 & 0.35 & 0 & 0 & -0.14 & 0 & 0 & 0.27 & 0.41 & 0 \\ 0 & 0 & 0 & 0 & 0 & 0 & 0 & 0 & 0 & 0 \\ 0 & 0.48 & 0 & 0 & 0 & 0 & 0 & -0.11 & 0 & 0 \\ 0.58 & 0 & -0.1 & 0 & 0 & 0 & 0 & 0 & 0 & 0 \\ 0 & 0 & 0.78 & -0.23 & 0 & 0 & 0 & 0 & -0.87 & 0 \\ 0 & 0 & 0 & 0 & 0 & 0.55 & 0 & 0 & 0 & 0 \\ 0 & 0 & 0 & 0 & 0.58 & 0 & 0 & 0 & -0.76 & 0 \\ 0 & -0.16 & 0 & 0 & 0 & -0.55 & 0 & 0 & 0 & 0.93 \\ 0 & 0 & 0 & 0.08 & 0 & 0 & 0 & 0 & 0 & -0.48 \\ 0 & 0 & 0 & 0 & 1 & 0 & 0 & 0 & -0.49 & 0 \end{matrix} \right]$$

(c) 10 nodes, density=20%

$$\left[ \begin{matrix} 0 & 0 & 0 & 0 & 0.59 & 0 & 0 & 0 & 0 & 0.35 \\ 0 & -0.41 & 0 & 0 & 0 & 0 & 0 & -0.96 & -0.76 & -0.22 \\ 0 & -0.23 & 0 & -0.6 & -0.29 & 0.25 & 0 & 0 & 0 & 0 \\ 1 & 0 & 0.75 & -0.8 & 0 & -0.39 & 0 & 0 & -0.6 & 0 \\ 0 & 0 & 0 & -0.63 & 0 & 0.50 & 0 & 0 & 0 & 0 \\ 0 & 0 & 0 & 0 & 0 & 0 & 0 & -0.73 & 0 & 0 \\ 0 & 0 & 0.81 & 0 & 0.27 & 0 & -0.07 & 0.42 & 0 & 0 \\ 0 & 0 & 0 & 0 & 0 & 0 & 0 & 0 & -0.28 & 0 \\ 0.69 & 0 & -0.77 & 0.15 & -0.09 & 0.34 & 0.22 & 0.23 & -0.71 & -0.85 \\ -0.7 & 0 & 0.21 & 0.11 & -0.18 & 0.39 & 0.09 & 0 & -0.94 & 0 \end{matrix} \right]$$

(d) 10 nodes, density=40%
